# Magnetic Porous Controlled Fe_3_O_4_–Chitosan Nanostructure: An Ecofriendly Adsorbent for Efficient Removal of Azo Dyes

**DOI:** 10.3390/nano10061194

**Published:** 2020-06-19

**Authors:** Tiago M. Freire, Lillian M. U. D. Fechine, Danilo C. Queiroz, Rafael M. Freire, Juliano C. Denardin, Nágila M. P. S. Ricardo, Thaina N. B. Rodrigues, Diego R. Gondim, Ivanildo J. S. Junior, Pierre B. A. Fechine

**Affiliations:** 1 Group of Chemistry of Advanced Materials (GQMat)—Department of Analytical Chemistry and Physical-Chemistry, Federal University of Ceará—UFC, Campus do Pici, CP 12100, Fortaleza CEP 60451-970, Brazil; tiagomf@ufc.br (T.M.F.); lmudutra@hotmail.com (L.M.U.D.F.); 2 Department of Organic and Inorganic Chemistry, Federal University of Ceará—UFC, Campus do Pici, CP 12100, Fortaleza CEP 60451-970, Brazil; daniloqueiroz46@gmail.com (D.C.Q.); naricard@ufc.br (N.M.P.S.R.); 3 Institute of Applied Chemical Sciences, Universidad Autónoma de Chile, Santiago 8910060, Chile; rafael.m.freire@gmail.com; 4 Department of Physical/CEDENNA, University of Santiago de Chile, USACH, Av. Ecuador 3493, Santiago 9170020, Chile; juliano.denardin@usach.cl; 5 Department of Chemical Engineering, Federal University of Ceará—UFC, Campus do Pici, CP 12100, Fortaleza CEP 60451-970, Brazil; thaina.nobre@hotmail.com (T.N.B.R.); diegoromao19@yahoo.com.br (D.R.G.); ivanildo@gpsa.ufc.br (I.J.S.J.)

**Keywords:** magnetite, chitosan, superparamagnetic nanocomposite, azo dyes

## Abstract

In this work, chitosan/magnetite nanoparticles (ChM) were quickly synthesized according to our previous report based on co-precipitation reaction under ultrasound (US) irradiation. Besides ChM was in-depth structurally characterized, showing a crystalline phase corresponding to magnetite and presenting a spheric morphology, a “nanorod”-type morphology was also obtained after increasing reaction time for eight minutes. Successfully, both morphologies presented a nanoscale range with an average particle size of approximately 5–30 nm, providing a superparamagnetic behavior with saturation magnetization ranging from 44 to 57 emu·g^−1^. As ChM nanocomposites have shown great versatility considering their properties, we proposed a comparative study using three different amine-based nanoparticles, non-surface-modified and surface-modified, for removal of azo dyes from aqueous solutions. From nitrogen adsorption–desorption isotherm results, the surface-modified ChMs increased the specific surface area and pore size. Additionally, the adsorption of anionic azo dyes (reactive black 5 (RB5) and methyl orange (MO)) on nanocomposites surface was pH-dependent, where surface-modified samples presented a better response under pH 4 and non-modified one under pH 8. Indeed, adsorption capacity results also showed different adsorption mechanisms, molecular size effect and electrostatic attraction, for unmodified and modified ChMs, respectively. Herein, considering all results and nanocomposite-type structure, ChM nanoparticles seem to be a suitable potential alternative for conventional anionic dyes adsorbents, as well as both primary materials source, chitosan and magnetite, are costless and easily supplied.

## 1. Introduction

As the global population is steadily growing, worldwide demand for textile products is also increasing. However, not only the textile industry is facing several environmental impacts—mainly related to wastewater discharged from textile dyeing processes. New efforts must be made to overcome this environmental issue [1]. Dyes are widely used in textile, paper and plastic industries and present high water solubility, where around 10–20% of these chemical compounds still remaining in the wastewater [2,3]. Furthermore, since mostly dyes are classified as toxic and carcinogenic substances [4], the environmental impact also reaches the safe use category considering human health.

Herein, considering the excessive dyes use, numerous dye-removal methods have been developed such as adsorption [5], heterogeneous Fenton process [6], photocatalytic degradation [7], coagulation–flocculation [8] and ultra-filtration through fine membranes [9]. Currently, adsorption method has been widely applied to remove different types of pollutants from effluents, due to their low cost and simplicity of design [10,11]. However, most textile industries have been used activated carbon as adsorbent, even presenting high cost, once can remove heavy metals and dyes with high adsorption capacity [12,13].

Recently, the scientific community has directed some advanced researches in the development of alternative adsorbents, especially those based on polymers [14,15]. Chitosan (β-(1,4)-2-amino-2-deoxy-D-glucose), a linear polysaccharide obtained from deacetylation of chitin, has been used as an efficient adsorbent of azo dyes [16]. Actually, chitosan and its derivates have shown as an outstanding material for adsorption of heavy metal ions [17] and organic dyes [18], principally due to their high content of amino (–NH_2_) and hydroxyl (–OH) groups on polymer skeleton. Additionally, this macromolecule has been considered ecofriendly, nontoxic and biodegradable [19,20,21]. However, chitosan water solubility is limited by acid medium, herein, some studies evidence the better chemical stability of chitosan being related to crosslinking reactions, using both amino and hydroxyl-based crosslinking agents such as epichlorohydrin [22], glutaraldehyde [23] and tripolyphosphate [11].

Therefore, considering aforementioned issues, magnetic chitosan-based nanoparticles have been used as an efficient adsorbent of organic dyes, since present great adsorption properties such as high separation efficiency, good relationship of cost-effectiveness and simple operation process [24]. In the literature, several methods have been applied to synthesize magnetic chitosan nanoparticles (ChM NPs), such as co-precipitation [25], reduction–precipitation [24] and dispersion in a polymer matrix [2]. However, these methods usually require a long reaction time and the Fe_3_O_4_ is not well-dispersed into polymer matrix [26]. Recently, our research group developed a fast ultrasound-assisted synthesis of ChM NPs using one quick step, which takes only two minutes, providing well-dispersed magnetite NPs into chitosan matrix with superparamagnetic behavior and average particle size around 10–24 nm [27].

Herein, in the present work, we propose to synthesize Fe_3_O_4_–chitosan nanocomposite NPs using previous reported US irradiation approach, with a step further of surface-modification, in order to investigate their behavior and properties regarding adsorption capacity and anionic azo dyes removal from aqueous dispersion. After whole structural and magnetic characterizations, reactive black 5 (RB5) and methyl orange (MO) were used as model of anionic dyes to evaluate the adsorption profile of ChMs. Indeed, operation and medium factors, including pH, contact time and dye concentration were also investigated. Furthermore, kinetic and isotherm models were applied to experimental data of RB5 and MO adsorption on Fe_3_O_4_–chitosan nanocomposite NPs.

## 2. Materials and Methods

### 2.1. Materials

**Figure 1:**
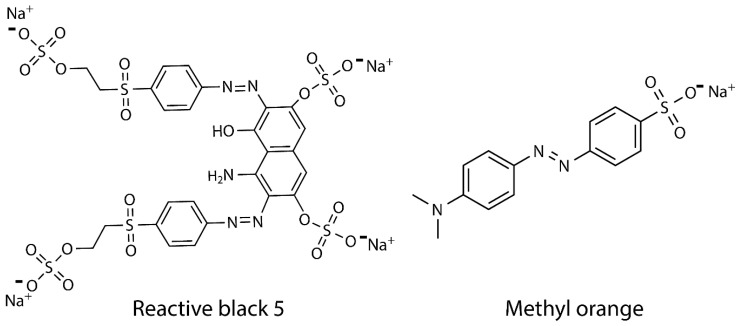
Chemical structure of anionic azo dyes reactive black 5 (RB5) and methyl orange (MO).

Ferric chloride hexahydrate (Fe_3_Cl∙6H_2_O, 97%) and glacial acetic acid (CH_3_COOH, 99.7%) were purchased from Vetec Ltd., Rio de Janeiro—RJ, Brazil. Chitosan was provided by DNP—Delta Produtos Naturais Ltd., Parnaíba - PI, Brazil (Molecular weight (M_w_) = 31 kDa and deacetylation degree (DD) = 92%). Ferrous sulfate heptahydrate (FeSO_4_∙7H_2_O, 99%) and ammonium hydroxide (NH_4_OH, 30%) were purchased from Dinâmica Ltd., Indaituba—SP, Brazil. Glutaraldehyde solution (25%) and epichlorohydrin (99%) were purchased from Sigma-Aldrich, Jurubatuba—SP, Brazil. The dyes, reactive black 5 (RB5, M_w_ = 991.82 g·mol^−1^) and methyl orange (MO, M_w_ = 327.34 g·mol^−1^) (Figure 1) were purchased from Sigma-Aldrich, Jurubatuba—SP, Brazil. All reagents are analytical grade and used as received.

### 2.2. Synthesis of Chitosan/Fe_3_O_4_ NPs

Chitosan/Fe_3_O_4_ NPs were synthesized following our previous published US method [27]. Briefly, 0.05 g of chitosan was dissolved in 15 mL of acetic acid 1% (*v/v*) under stirring for 10 min. Then, 10 mL of Fe 0.33 mol·L^−1^ (FeCl_3_·6H_2_O:FeSO_4_·7H_2_O, 2:1 molar ratio) solution was added, and the obtained mixture was homogenized using an US probe for 4 min (20 kHz, 585 W). Under US irradiation, 2 mL of NH_4_OH was slowly added and the system was kept under sonication for more 4 min. Finally, the obtained powder was magnetically decanted and washed several times with distilled water, and further dried under vacuum for 24 h. The synthesized material was so-called ChM.

### 2.3. Preparation of Surface-Modified Chitosan/Fe_3_O_4_ NPs

Initially, ChM was synthesized and purified as previously described, and then dispersed into crosslinking agent solutions, GL (chitosan:GL, 2:1 molar ratio) and ECH (chitosan:ECH, 2:1 molar ratio, pH = 10). The dispersion was maintained under mechanical stirring at 50 °C for 2 h. The ChM NPs were washed several times with distilled water and dried under vacuum for 48 h. The surface-modified NPs were labeled as ChM GL and ChM ECH.

### 2.4. Characterization Methods

X-ray powder diffraction (XRD) analysis was performed to confirm the crystalline structures of magnetite present in chitosan/Fe_3_O_4_ NPs. All samples were analyzed by X-ray powder diffractometer Xpert Pro MPD (Panalytical, São Paulo—SP, Brazil) using Bragg–Brentano geometry in the range of 15–70° with a rate of 1°·min^−1^. The samples Fe_3_O_4_, ChM GL and ChM ECH were analyzed using CuKα radiation (k = 1.54059 Å) and the tube operated at 40 kV and 30 mA, whereas ChM sample was analyzed using CoKα radiation (k = 1.78896 Å) and the tube operated at 40 kV and 30 mA.

Fourier-transform infrared spectroscopy (FTIR) analyses were carried out in a PerkinElmer 2000 spectrophotometer used to record spectra in the range 4000–400 cm^−1^. Previously, the samples were dried and grounded to powder and pressed (~10 mg of the sample to 100 mg of KBr) in a disk format (KBr pellet).

The micrographs were obtained using a HITACHI HT7700 transmission electron microscope (TEM) (HITACHI, Santiago, Chile) operating at accelerating voltage of 120 kV. Before analysis, the samples were dispersed in water and one droplet was placed on 300 mesh carbon-coated copper grid and dried overnight under ambient conditions.

Thermogravimetric analysis (TGA) was carried out under a nitrogen atmosphere using a Thermogravimetric Analyzer Q50 (Shimadzu, Barueri—SP, Brasil). The weight loss (%) was monitored by heating the samples from 25 to 850 C with a rate of 10 C·min^−1^. The zero time for the thermal degradation study was taken after temperature stabilization. Derivative thermogravimetry (DTG) was obtained by first derivative of respective TGA curves.

K-Alpha X-ray photoelectron spectrometer (XPS) (Thermo Fisher Scientific, Renfrew, United Kingdom), equipped with a hemispherical electron analyzer and an aluminum anode (Kα = 1486.6 eV), was used to determine the chemical surface composition of the nanocomposites. All measurements were carried out using charge compensation during analyses, and the pressure of the chamber was kept below 2 × 10^−8^ mbar. Survey (i.e., full-range) and high-resolution spectra were recorded using pass energies of 1 and 0.1 eV, respectively. The spectrum fitting was performed by the Shirley method, assuming a mixed Gaussian/Lorentzian peak shape with the ratio of Gaussian to Lorentzian fixed in 0.4. In this work, X-ray photoelectron spectra are a result of the average of three spectra collected at three different regions.

Magnetic properties were investigated by a vibrating sample magnetometer (VSM) Mini 5 Tesla from Cryogenic, Ltd, London, United Kingdom. Previously, the VSM was calibrated using a YIG sphere, and after measuring the mass of each sample, the magnetization was given in emu·g^−1^.

N_2_ adsorption/desorption experiments at 77 K were carried out using a volumetric adsorption equipment (Autosorb 1c, Quantachrome, Boynton Beach, FL, USA and Micromeritics, ASAP 2000, Norcross, GA, USA). The specific surface area (S_BET_) of nanocomposites was estimated according to Brunauer−Emmett−Teller method. The pore size distribution, average pore diameter and total pore volume were calculated from density functional theory (DFT) method. All samples were previously degassed at 100 °C.

### 2.5. Adsorption Assay

Adsorption experiments were performed in triplicate in a rotary shaker system at 18 rpm and 25 °C (±1 °C). For all samples, the amount of adsorbent and volume of dye solution, RB5 and MO, were kept constant, 10 mg and 3 mL, respectively.

#### 2.5.1. pH Effect

For all performed experiments, the concentration of RB5 and MO was 100 mg·L^−1^, which was analyzed at four different pH values (4, 6, 8 and 10), in order to investigate the effect of the pH level in the adsorption capacity of nanocomposites. First, a stock solution of each dye was prepared using distilled water. Then, 50 mL of the prepared stock solution was added in a glass flask, and further HCl and/or NaOH solution were added to adjust pH level of the medium. The samples were kept for 2 h under shaking in a rotary shaker. The residual concentration of the dyes was determined using a UV spectrophotometer at 597 and 505 nm for RB5 and MO, respectively. For dye amount determination (*q_e_* in mg·g^−1^) was applied the following equation:

(1) $$q_{e}=\frac{V_{Sol}\left(C_{0}-C_{eq}\right)}{m_{ads}}$$

where *C*_0_ (mg·L^−1^) represents the initial dye concentration, *C_eq_* (mg·L^−1^) is the equilibrium concentration of the dye remaining into solution, *V_Sol_* (L) is the volume of aqueous solution and *m_ads_* (g) is the mass of used adsorbent.

#### 2.5.2. Adsorption Kinetics

Kinetics experiments were performed by mixing 10 mg of the adsorbent and 3 mL of dye solution (pH 4, *C*_0_ = 100 mg·L^−1^). The adsorbent aliquots were collected at different fixed times, 1, 5, 10, 20, 40, 60, 90, 120, 150 and 180 min and then removed by magnetic decantation. The pseudo-first-order (PFO) and pseudo-second–order (PSO) models were selected to fit the experimental kinetic data [28,29]. These models assume that the adsorption is a pseudo-chemical reaction, where the adsorption rate can be determined using the following equations:

Pseudo-first order

(2) $$q_{t}=q_{e}\left(1-\exp\left(k_{1}t\right)\right)$$

Pseudo-second order

(3) $$q_{t}=\frac{t}{\left(\frac{1}{k_{2}q_{e}^{2}}\right)+\left(\frac{1}{q_{e}}\right)}$$

where *k*_1_ and *k*_2_ are kinetic coefficients of the pseudo-first and second–order (min^−1^ and g·mg^−1^·min^−1^), respectively, and *q_e_* is the theoretical values for adsorption capacity (mg·g^−1^).

The intraparticle diffusion model was used once PFO and PSO cannot determine the diffusion mechanism. The initial rate of intraparticle diffusion model is obtained by the Equation (4):

(4) $$Q_{t}=K_{d}\cdot t^{0.5}+C$$

where $K_{d}$ is the intraparticle rate (g·mg^−1^·min^−5^) and *C* is a constant. In general, the intraparticle plots should show three regions assigned to: (i) instantaneous adsorption, which is influenced by external mass transfer of the adsorbent; (ii) internal diffusion and (iii) equilibrium condition [10].

#### 2.5.3. Adsorption Isotherms

Adsorption equilibrium isotherms are very important to describe the interactive behavior between adsorbate and adsorbent, which it is fundamental in the design of an adsorption method. Therefore, isotherm assays were performed adding 10 mg of adsorbent into different systems, varying dye concentration from 25 to 300 mg L^−1^ and keeping the acid medium at pH 4. Various isotherm models such as Langmuir [30], Freundlich [31], Redlich–Peterson [32] and Temkin [33,34] were used to analyze the experimental data. In general, these models are used to describe the adsorption process of dyes on chitosan-based materials [35]. The Langmuir isotherm considers that adsorption takes place at specific homogeneous sites onto adsorbent, and after adsorption in a specific site, no further adsorption can occur on the same site. In addition, the rate of adsorption capacity should be proportional to concentration of the adsorbate and specific surface area of the adsorbent. The equation of Langmuir isotherm is written below:

(5) $$q_{e}=\frac{q_{m}K_{a}C_{e}}{1+K_{a}C_{e}}$$

where $K_{a}$ (L·mg^−1^) is a constant related to the affinity of binding sites and *q_m_* represents the maximum adsorption capacity of the material (mg·g^−1^), assuming a monolayer of adsorbate on the adsorbent surface; *C_e_* (mg·L^−1^) is the adsorbate concentration at equilibrium condition of adsorption, and *q_e_* is the amount of dye per unit of mass of composite (mg·g^−1^). The main characteristic of Langmuir isotherm can be expressed in terms of both equilibrium and dimensionless constant, called separation factor *R_L_*, which can be rewritten by the following equation [3,36]:

(6) $$R_{L}=\frac{1}{1+K_{a}C_{0}}$$

where *C*_0_ (mg·L^−1^) is the initial concentration of the dyes. According to the values of *R_L_*, the type of isotherm can be interpreted as unfavorable (*R_L_* > 1), linear (*R_L_* = 1), favorable (0 < *R_L_* < 1) and irreversible (*R_L_* < 0). As shown in Figure S1 (Supplementary Materials), the values of *R_L_* calculated in this work are between 0 and 1, indicating that the adsorption of both dyes are favorable, considering concentration range.

The Freundlich isotherm model is an empirical equation, which assumes another adsorption process, heterogeneous surface, where multilayers of the adsorbate is formed onto adsorbent. The model can be described according to Equation (7).

(7) qe=kFCe1n

where *k_F_* (L·g^−1^) is the Freundlich constant defined as adsorption of the distribution coefficient and represents the quantity of dye adsorbed onto adsorbent at equilibrium concentration. The parameter *n* is the Freundlich exponent (dimensionless) related to surface heterogeneity. In addition, for a favorable adsorption process, the value of 1/*n* should be between 0 and 1 [37].

The Redlich–Peterson isotherm incorporates the features of Langmuir and Freundlich isotherms. Thereby, the isotherm model can be applied in both homogeneous and heterogeneous systems and also used to represent adsorption equilibrium over a wide concentration range. In addition, this model assumes that monolayer and multi-sites adsorptions is occurring concomitantly. The Redlich–Peterson isotherm is given by Equation (8) [38].

(8) $$q_{e}=\frac{K_{RP}C_{e}}{1+A_{RP}C_{e}^{\beta }}$$

where *C_e_* is dye concentration in equilibrium, *K_RP_* and *A_RP_* are the Redlich–Peterson constants (L·mg^−1^) and (L·mg^−1^)*^β^*, respectively. β is the exponent ranging between 1 and 0, and when *β* → 1 this model tends to Langmuir model, i.e., isotherms at low concentration. Instead, when *β* → 0, isotherm tends to Freundlich model at high concentration [39].

The Temkin isotherm model considers adsorbent-adsorbate interactions, assuming that the heat of adsorption in the layer decreases linearly with coverage, in which can occur with a uniform binding energy distribution up to some maximum binding energy [40], as described by Equations (9) and (10).

(9) $$q_{e}=Bln\left(K_{T}C_{e}\right)$$

(10) $$B=\frac{RT}{b}$$

where $C_{e}$ is the equilibrium concentration of the dye, T is the temperature, R (8.314 J·mol·K^−1^) is the universal gas constant, $K_{T}$ (L·mg^−1^) is the equilibrium binding constant, corresponding to the maximum binding energy, and B (J·mol^−1^) is the constant related to the heat of adsorption.

## 3. Results and Discussion

### 3.1. Structure and Morphology Analyses

#### 3.1.1. X-ray Powder Diffraction

**Figure 2:**
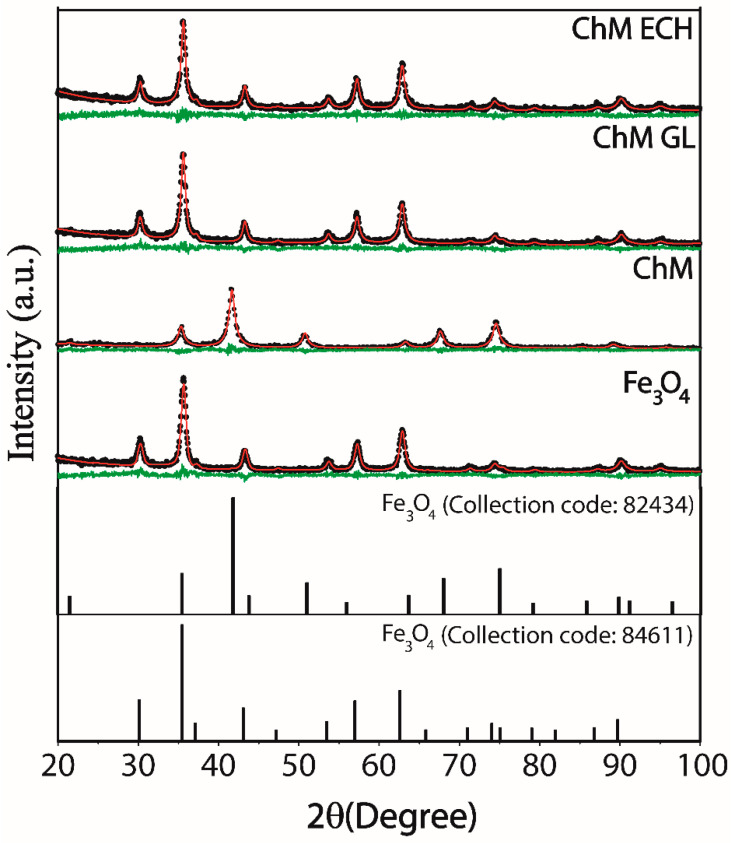
X-ray powder diffraction (XRD) patterns of standard Fe_3_O_4_ (ICSD (Inorganic Crystal Structure Database), file n° 01-086-1340, standard Fe_3_O_4_ (ICSD, file n° 01–086-1340), Fe_3_O_4_, chitosan-magnetite nanoparticles (ChM) ChM GL and ChM ECH. Green lines is the difference between the observed (black dots—I_Obs_) and the calculated (red line—I_Calc_) intensities from the Rietveld method.

XRD was used to confirm the magnetic crystalline structure present in the nanocomposite, and also to verify if the post-modification step could change crystalline structure of the final material. The profile diffraction peaks of the cubic spinel phase of magnetite (Fe_3_O_4_) can be observed in all synthesized samples, as shown in Figure 2, where the pattern diffraction peaks are associated with (220), (311), (400), (422), (511), (440) and (533) crystal planes of the Fe_3_O_4_ (ICSD, file n° 01-086-1340 and ICSD, file n° 01-086-1340). In general, it was possible to notice that the adopted post-modification methodology does not change the crystalline structure of ChM NPs. Indeed, as expected, the synthesis strategy based on co-precipitation reaction of the iron–chitosan complex under US irradiation provides chitosan-coating magnetite NPs, promoting resistance against oxidation. Moreover, the US irradiation plays a significant rule in the synthesis, obtaining a quick healing and a homogeneous dispersion material. In contrast, Jing-Jing Cui and coworkers [41] synthesized surface-modified magnetite–chitosan nanoparticles with thiosemicarbazide, and observed changes in the magnetite crystalline structure, suggesting a non-efficient chitosan coating.

The diffractograms were refined by Rietveld method and the obtained parameters such as weighted profile R-factor (R_wp_), goodness of fit index (χ^2^), lattice parameters (a, b and c) and diameter crystallite size (D) with its respective standard deviation values, were summarized in Table 1. The R_wp_ and χ^2^ are parameters used to verify the agreement between calculated and experimental data. For our samples, low values of R_wp_ and χ^2^ were observed, suggesting goodness of the Rietveld refinement. Furthermore, after refinement, Equation (11) (Debye–Scherrer equation) was used to estimate the crystallite size of MNPs.

(11) $$D=\frac{k\lambda }{\beta Cos\left(\theta \right)}$$

where k is the shape coefficient, λ is the X-ray wavelength, β is the full width at half of the maximum intensity and θ is Bragg’s angle. In Table 1, it was no further observed a significant change in the crystallite size and lattice parameters related to functionalization with chitosan. However, after crosslinking reaction, a small contraction of the unit cell of magnetite was observed, indicating a possible surface passivation effect [5].

#### 3.1.2. Fourier-Transform Infrared Spectroscopy (FTIR)

**Figure 3:**
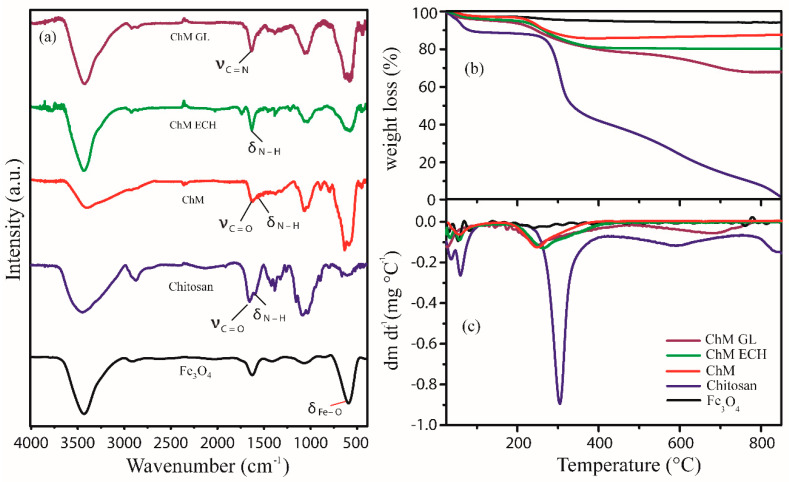
(**a**) Fourier-transform infrared spectroscopy (FTIR) spectra, (**b**) Thermogravimetric analysis (TGA) and (**c**) Derivative thermogravimetry (DTG) of the samples Fe_3_O_4_, chitosan, ChM, ChM ECH and ChM GL.

FTIR was performed to provide the structural profile of nanocomposites. Figure 3a shows FTIR spectra of Fe_3_O_4_, chitosan, ChM, ChM ECH and ChM GL, and for all analyzed samples, excepted for chitosan, the bands around 586 and 634 cm^−1^ were assigned to lattice vibrations of Fe–O in tetrahedral and octahedral positions, confirming the presence of Fe_3_O_4_ into nanocomposite structure [42]. For chitosan spectrum, the bands at 1027, 1083 and 1155 cm^−1^ can be related to stretching vibration C–O of ether, stretching vibration of primary alcohol and special adsorption of β (1→4) glucoside bonds, respectively [43]. These bands were also observed in nanocomposite spectra, however, showing a slightly displacement. The bands at 1326, 1382 and 1423 cm^−1^ can be referred to axial deformation C–N of amino groups, and bending vibration of methylene and methyl groups, respectively [44,45]. Interestingly, in nanocomposite spectra, these bands show a different format, as well as the band at 1326 cm^−1^ is displaced to 1316, 1311 and 1315 cm^−1^ for samples ChM, ChM GL and ChM ECH, respectively.

For chitosan spectrum, two bands at 1595 and 1654 cm^−1^ were assigned to N–H bending and C=O–stretching vibrational bands, respectively. In ChM sample, these bands arise at lower wavelengths at 1530 and 1621 cm^−1^, respectively. This displacement may be assigned to the chelation effect of chitosan molecules with iron ions during formation of the nanocomposite, suggesting that Fe atoms on magnetite surface is coordinated to amino groups from chitosan [46,47]. For ChM ECH sample, no further evidence was found regarding crosslinking reaction between chitosan and ECH, since any different band was observed in comparison to chitosan and magnetite spectra. However, in ChM GL spectrum, the surface-modification with GL is evidenced at 1631 cm^−1^, which can be assigned to stretching vibration of C=N from imine groups, i.e., Schiff base linkages. Additionally, no band was observed in the range of 1725 cm^−1^, suggesting that aldehyde groups also reacted with chitosan molecules on nanocomposites surface [48].

#### 3.1.3. TGA–DTG

TGA was used to evaluate thermal stability of nanocomposites. Figure 3b,c shows TGA and DTG curves for Fe_3_O_4_, chitosan and ChM samples, respectively. For all samples, a first event occurs at a temperature range of 30–130 °C, which can be assigned to residual water loss. The hydration of polysaccharides depends on their primary and supramolecular structure, thus, the first event may provide information about the physical and molecular changes caused by the interactions of magnetite and crosslinking agents [49]. Therefore, for pure chitosan, it was also verified a first stage of residual water loss of 12%, whereas for ChM was approximately 3%. For pure chitosan, DTG curve shows two events at 36 and 60 °C that can be assigned to the breakdown interaction of water molecules with amino and hydroxyl groups [50,51]. For all ChM samples, considering initial events, both amino and hydroxyl groups must interact with magnetite surface, becoming less available to interact with water molecules. This profile was also supported by ChM DTG curves, showing just one event at 56 °C related to water loss [49]. For ChM GL and ECH, the first stage of residual water loss was around 5% and 6%, respectively—and from DTG curves—it was also possible to notice a similar profile of chitosan and ChM ECH, which initial events can be also assigned to residual water loss. However, for ChM ECH, these events appear displaced to lower temperature due to the crosslinking agent ECH, since the post-modification step can occur in both amino and hydroxyl groups, converting hydroxyl in ether groups and forming a dense crosslinked network [52]. Instead, for ChM GL, the crosslinking reaction converts primary amino to imine groups. Then, water molecules may interact with hydroxyl groups and, consequently, the dehydration process can be considered more difficult [50].

The second event occurs in the range of 228–430 °C for chitosan and 155–480 °C for all nanocomposites, corresponding to polymer degradation [53], suggesting that covalent binding with GL and ECH on nanocomposite surface decreases chitosan thermal stability. Indeed, this may correspond to the cooperative loss of the hydrogen bond between chitosan chains, due to interactions between amino and hydroxyl groups from crosslinking agent molecules with magnetite surface [54]. In DTG curves, considering second thermal event, it was also noticed a lower rate of weight loss for modified nanocomposites, 47.8%, 11.0%, 16.8% and 15.0% for chitosan, ChM, ChM GL and ChM ECH, respectively. For chitosan and ChM GL samples, the thermal degradation continues until 870 °C, and may be related to the breakdown of chitosan chains on magnetite surface.

#### 3.1.4. X-ray Photoelectron Spectroscopy (XPS)

**Figure 4:**
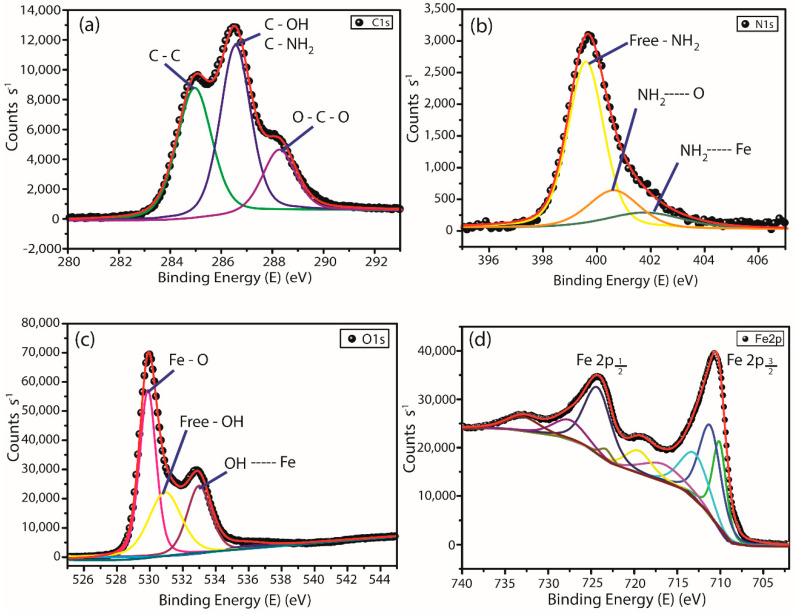
High resolution X-ray photoelectron spectroscopy spectra of (**a**) C 1s, (**b**) N 1s, (**c**) O 1s and (**d**) Fe 2p of the sample ChM.

XPS spectra could provide interaction information between adsorption sites from chitosan and magnetite. Additionally, XPS was used to determine the valence state of iron and the sites into magnetite structure. Furthermore, since DRX results showed no further change in the magnetite crystalline structure after post-modification step, ChM was chosen as a representative sample for XPS analysis. Herein, Figure 4 only shows spectra regarding ChM sample.

The best fit for C 1s, O 1s and N 1s spectra was obtained with three components, whereas for Fe 2p spectrum was nine components. Table 2 summarizes the results obtained from deconvolution of the peaks observed in all spectra for ChM sample.

None of the peaks observed in the C 1s spectrum are related to the interactions with iron ions. For N 1s, two peaks arise from the contribution of three components at 399.6, 400.6 and 402.0 eV, which can be attributed to free amino groups (NH_2_), where some amino groups are also involved in hydrogen bonds (NH_2_---O) and chelated with iron ions (NH_2_---Fe). Similar profile was observed for O 1s spectrum, where the components at 529.8 and 530.9 eV may be attributed to the oxygen linkage with iron ions and free hydroxyl groups, respectively. However, the component at 533.0 eV can be assigned to hydroxyl groups in hydrogen bonds (–OH---O and–OH---N) and also chelated with iron ions (–OH---Fe). It is interesting to notice that the binding energy attributed to hydroxyl groups and iron ions may be due to both water adsorption on magnetite surface and chitosan hydroxyl groups.

In Fe 2p spectrum, as seen in Figure 4d, it was observed a doublet Fe 2p3/2 at 710.6 eV and Fe 2p1/2 at 724.2 eV regarding to Fe^2+^ octahedrally and Fe^3+^ octahedrally and tetrahedrally coordinated, which is characteristic of magnetite inverse spinel structure. Thus, these results suggest that both nitrogen and oxygen atoms were involved in the complex formation of iron–chitosan during ChM NPs synthesis. Wang and coworkers, which presented a more detailed XPS study about chitosan, chitosan—iron (II) and iron (III) complex and chitosan–Fe_3_O_4_, observed that the atomic fraction associated with hydroxyl groups, involved in hydrogen bonds, increases in the sample containing iron. In this regard, the authors suggested that this profile may be due to interaction between hydroxyl groups and iron ions (OH---Fe) [55]. Thus, in this work, it is possible to propose that both nitrogen and oxygen atoms participate in the complex formation of iron ions–chitosan.

**Figure 5:**
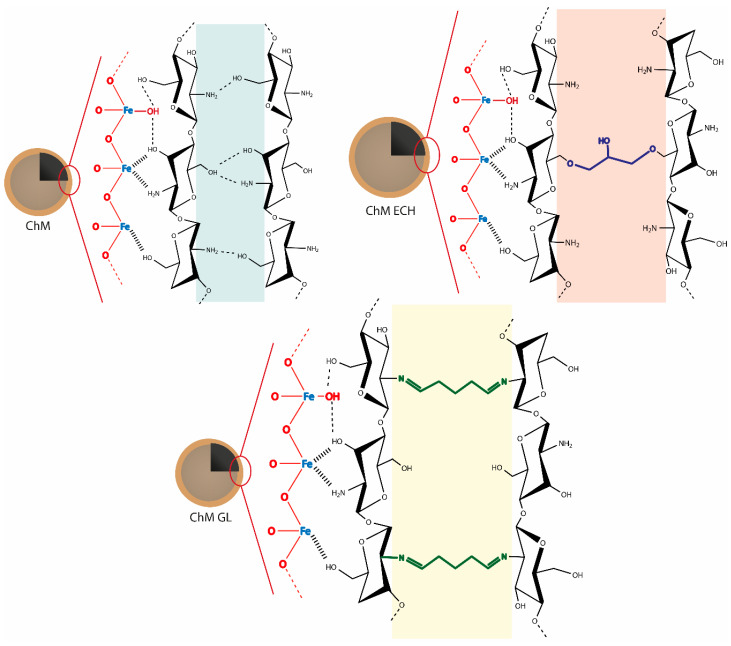
Proposal of surface structure of nanocomposites.

The results of XPS, FTIR, TGA and XRD are important to understand the type of crystalline structure is being formed after synthesis, as well as, the type of interaction between chitosan and magnetite surface. Additionally, it was also possible to verify that GL and ECH crosslinking agents could affect the intermolecular interactions in chitosan structure. Therefore, in Figure 5 is shown a schematic proposal of nanocomposites surface synthesized in this work. As can be seen, besides all nanocomposites show similar chitosan–iron complex formation on magnetite surface, for ChM GL and ECH, is also shown the crosslinked network between chitosan molecules on ChM NPs after post-modification step.

#### 3.1.5. TEM

**Figure 6:**
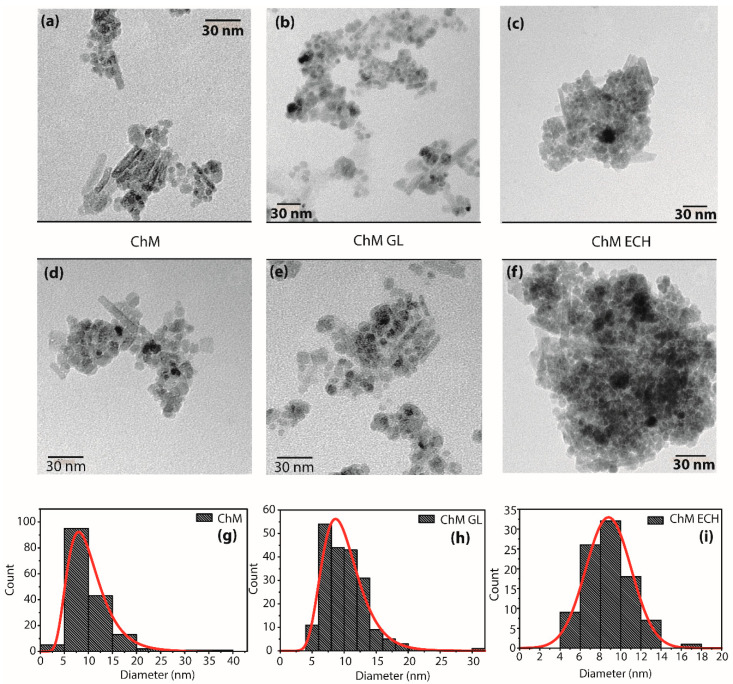
Transmission electron microscope (TEM) images of the samples ChM (**a**,**d**), ChM GL (**b**,**e**) and ChM ECH (**c**,**f**) at different amplifications. Histograms for spherical nanoparticles of the sample ChM (**g**), ChM GL (**h**) and ChM ECH (**i**), which were determined by measurement of 159, 201 and 93 randomly selected NPs at different regions of TEM images, respectively. Lognormal distribution curve was applied to describe the results.

TEM micrographs were used to investigate the morphology of the samples ChM, ChM GL and ChM ECH. As shown in Figure 6, the nanoparticles were successfully obtained in a nanoscale range, however, all samples had two distinct morphologies, rods and spheres, in which the rod particles show a heterogeneous length and diameter values. For spherical NPs, the histograms show an average diameter of 9.6 ± 3.4, 9.9 ± 3.1 and 8.7 ± 2.2 nm for ChM, ChM GL and ChM ECH, respectively (Figure 6g–i). It is interesting to mention that nanorods NPs were no further observed in our previous published work [27]. Indeed, in last years, various synthetic routes have been developed to synthesize 1D magnetite nanorods due to specific properties, such as unique electronic transport [39,56], and according to our knowledge, no study has been reported “one-pot” synthesis of magnetite–polymer nanorod using US irradiation.

For instance, Li and co-workers [57] reported that the increase in reaction time under US irradiation may cause a shape change of Se nanoparticles from spherical α–Se to t–Se nanotubes and further to t–Se nanowires. Neto and co-workers [58] have synthesized magnetite–polymers by US method using a longer reaction time in comparison to our method, instead the final nanoparticles did not present nanorods-type morphology. In addition, it is well-known that the capping agent can play an essential role in the preferential growth of a crystal, due to changes of the free energy of different facets [59]. Herein, we believe that the formation of chitosan/magnetite composite nanorods is dependent of both the US irradiation time increment and the capping agent chitosan. However, the appearance of nanorods shape will be deeper investigate in a further work.

### 3.2. Magnetic Property

**Figure 7:**
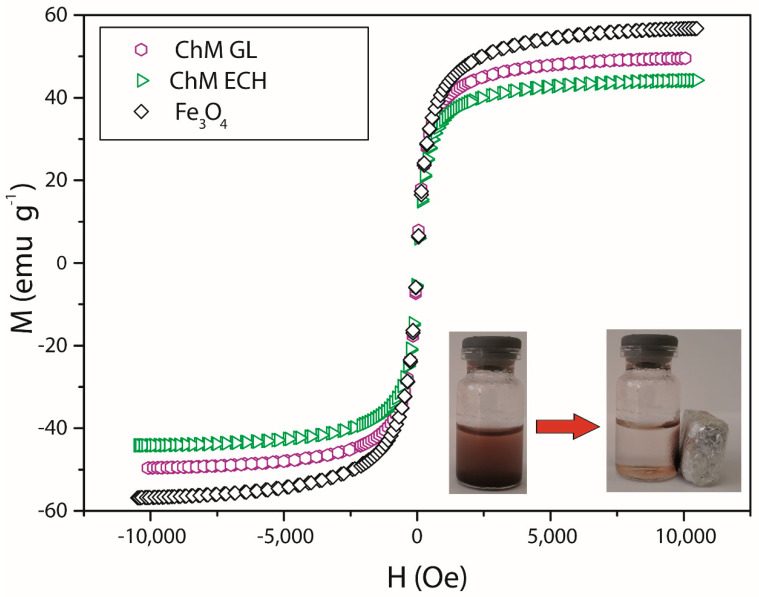
Magnetization curves normalized per gram of sample (emu.g^−1^).

In order to analyze magnetic behavior of synthesized GL and ECH-modified nanocomposites, the magnetization of the samples ChM GL and ChM ECH were measured as a function of the external magnetic field, and compared to magnetization curve of pure Fe_3_O_4_, using the same synthesis method. The results are shown in Figure 7. In addition, a photo (inset in Figure 7) of the sample ChM GL dispersed in water is shown, which presents the macroscopic magnetic behavior under an external magnetic field.

For all samples, an initial steep slope in magnetization curves is observed, suggesting that nanoparticles are small enough to be considered as single-domain particles [59]. The single-domain characteristic provides a data profile with almost no hysteresis loop, indicating that the samples have superparamagnetic behavior. For samples Fe_3_O_4_, ChM GL and ChM ECH, the saturation magnetization (M_s_) are found to be 56.78, 49.54 and 44.01 emu·g^−1^, respectively. Once the magnetization curve is a function of the material magnetization, MNPs functionalized with diamagnetic polymers trend to show smaller saturation magnetization values than unmodified ones. However, in this work, the obtained nanocomposites have great magnetic properties, even after surface-modification of ChM with glutaraldehyde and epichlorohydrin.

### 3.3. N_2_ Adsorption–Desorption

**Figure 8:**
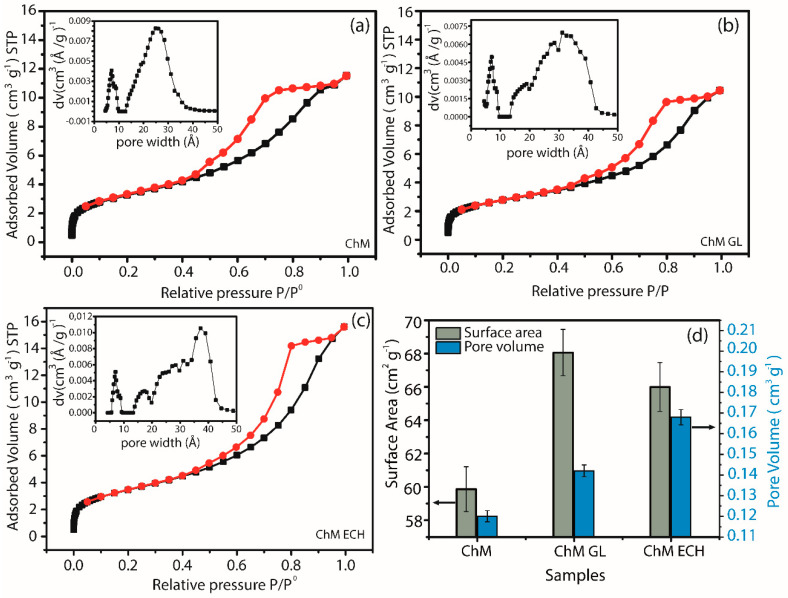
Nitrogen adsorption–desorption isotherms and pore size distribution of (**a**) ChM; (**b**) ChM GL and (**c**) ChM ECH; and (**d**) surface area and pore volume of nanocomposite samples.

N_2_ adsorption–desorption isotherms of nanocomposites and DFT method for pore size distribution (inset) are presented in Figure 8. The isotherms can be classified as type IV(a), according to IUPAC, which is related to pore size distribution in the range of mesopores (2 nm < d < 50 nm) [60]. Besides, for all samples, the isotherm hysteresis loop shows an H2(b)-type, which can be associated with materials with heterogeneous pore size, and generally with an ink-bottle shape pore. From the inset, it is also seen a change in the pore size distribution, which may be promoted by post-modification of chitosan with GL and ECH. For instance, Louis Poon reported that crosslinking reactions may decrease the porosity of final material, mainly due to the decrease in intraparticle diffusion occasioned by highest reaction rate on the surface of the polymer [54]. Thus, in this work, the formation of the ink-bottle shape pore can be due to the higher amount of crosslinking agent on the surface of nanocomposite. Figure 8d shows the Brunauer–Emmett–Teller (BET) surface area and pore volume for ChM, ChM GL and ChM ECH samples. The highest BET surface area was observed for the sample ChM GL, 68.0 m^2^ g^−1^, followed by ChM ECH and ChM with 66.0 and 59.8 m^2^ g^−1^, respectively. It is also important to notice that the pore volume increases for GL and ECH modified-nanocomposites. This behavior can be related to the decrease in intermolecular interactions among chitosan molecules, mainly occasioned by crosslinking reaction, in which was also evidenced by TGA/DTG curves.

### 3.4. Adsorption Evaluation

The adsorption capacity of the nanocomposites was evaluated using RB5 and MO as model anionic pollutants of textile industries. Indeed, the chosen dyes have shown some chemical characteristics as model molecules, since both azo (–N=N–) and sulfonic (SO_3_^−^) groups are found in various textile dyes [61].

#### 3.4.1. pH Effect

**Figure 9:**
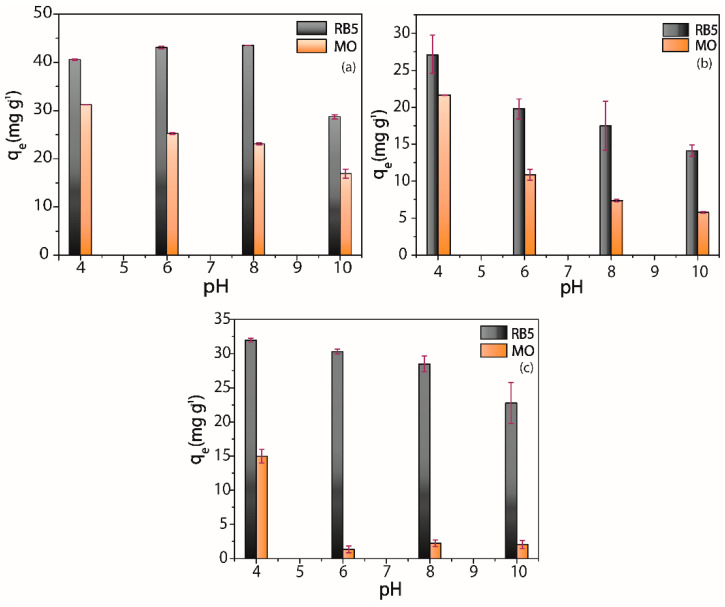
Adsorption capacity of RB5 and MO at different pH levels: (**a**) ChM, (**b**) ChM GL and (**c**) ChM ECH.

The pH level of dye solution plays an essential role in the adsorption process since can change the degree of interaction between adsorbent and adsorbate, directly affecting the adsorption capacity. Therefore, the effect of pH in RB5 and MO adsorption was investigated, and the results are shown in Figure 9a–c. For unmodified ChM sample, the adsorption profile was different for both MO and RB5 dyes, the increase of pH from 4 to 10 decreases the adsorbed amount of MO (Figure 9a). Interestingly, when the pH increases from 4 to 8, it was noticed a slightly increase in the amount of RB5 adsorbed, and further decreases when pH reaches 10. Generally, the change of the pH to acid medium increases the reactivity of dyes adsorption capacity of chitosan, which is related to protonation of primary amino groups, since protonated groups can interact through electrostatic attraction with anionic groups (–SO_3_^−^) in dye molecules [34,62]. Besides this behavior was observed for MO adsorption, the opposite was noticed for RB5 adsorption, suggesting that the electrostatic interaction does not act as a dominant adsorption mechanism. Additionally, the RB5 adsorption capacity decreases when pH value changes from 8 to 10, which may be related to the change of ChM MPs surface charge from positive to negative, since the pH zpc (point of zero charge) level was 8.91 (Figure S2, Supplementary Materials). Moreover, this decrease in the adsorption capacity could be due to competition between hydroxyl groups (OH^−^) and dye molecules in the reaction medium by adsorption active sites [21].

For ChM GL (Figure 9b) and ECH (Figure 9c) samples, it can be seen similar behavior in the adsorption capacity for both dyes, i.e., increasing the pH from 4 to 10 the adsorption capacity decreases. Tomasz and coworkers also observed the same adsorption profile using different reactive dyes for surface-modified chitosan samples [63], suggesting electrostatic interactions as the main dominant adsorption mechanism for both GL and ECH-modified nanocomposites synthesized in this work.

It is also seen that both dyes showed a higher adsorption capacity for ChM sample. For MO dye, all samples presented their maximum adsorbed capacity at pH 4. However, this profile was not observed for RB5 dye. For instance, ChM GL and ECH showed their maximum adsorbed amount (q_e(max)_), 27.06 and 31.96 mg g^−1^, respectively, at pH 4, whereas for ChM the q_e(max)_, 43.50 mg g^−1^, was obtained at pH 8. Besides the difference between q_e(max)_ values at pH 4 and 8 were quite small, pH 4 seems to be ideal for both dyes, considering all adsorption capacity values.

#### 3.4.2. Effect of Contact Time

**Figure 10:**
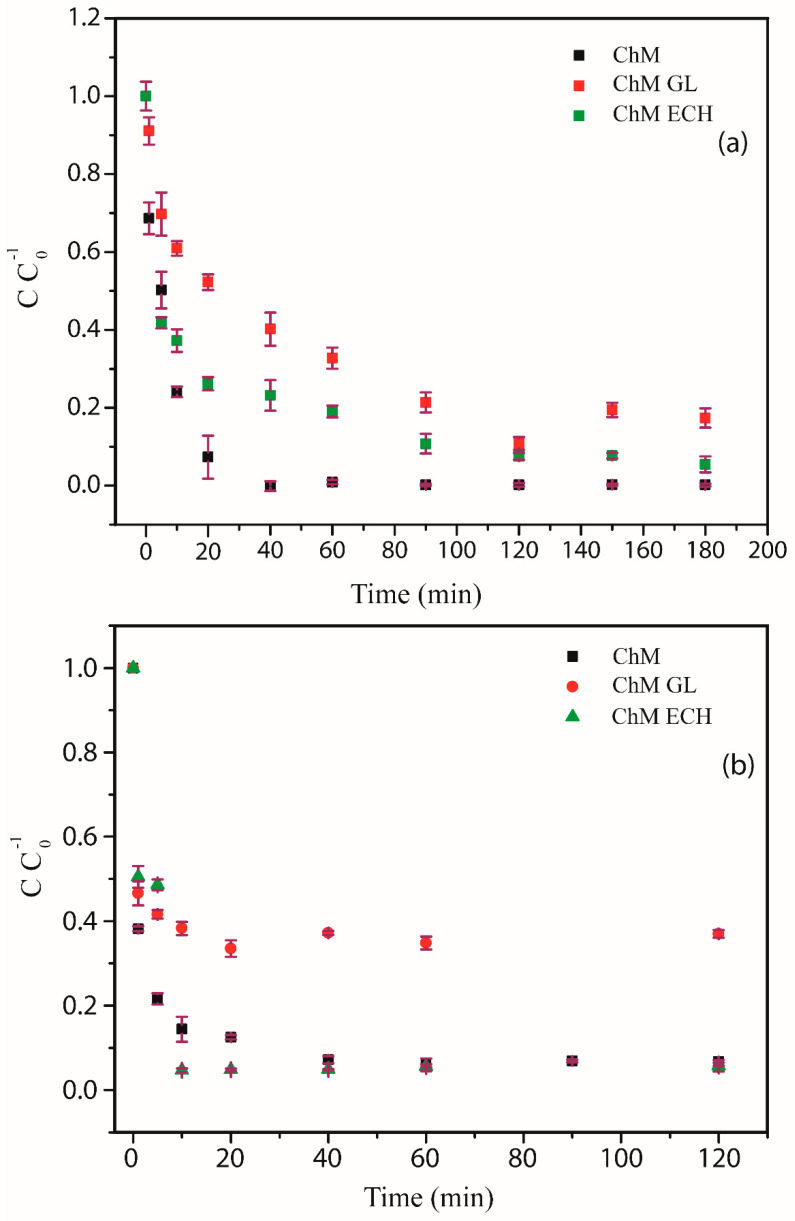
Adsorption kinetics of (**a**) RB5 and (**b**) MO of ChM, ChM GL and ChM ECH at pH 4.

Figure 10a,b show the change in the uptake of RB5 and MO dyes, respectively, by nanocomposites as a time function with dye initial concentration of 100 ppm and pH 4. For RB5, the sample ChM reaches the equilibrium condition at 40 min, whereas modified samples reach the equilibrium after 120 min. Indeed, after 40 min, ChM removed around 99.9% of the dye from acid solution and ChM ECH and GL—even at equilibrium condition—have only been removed 92.5% and 89.3% of the dye, respectively. For MO, the opposite behavior was observed, i.e., modified samples reach the equilibrium condition at 20 min, whereas ChM just after 40 min.

Munagapati and co-workers synthesized goethite–chitosan composite and verified an adsorption equilibrium time for MO is reached after 180 min, showing a slower adsorption profile in comparison to our samples [64]. Additionally, for ChM ECH and ChM samples at equilibrium condition, there was just a slight difference of the amount of removed dye, 94.3% and 93.2%, respectively. Indeed, adsorption kinetic may be explained mainly by two steps: (I) the initial section of the curve corresponds to a great availability of reactive groups and a large concentration gradient between solution and both surface and internal sorption sites; (II) the second step is controlled by a decrease of the concentration gradient and by resistance to intraparticle diffusion [65]. Aguiar has demonstrated that the adsorption of molecules with diameters lower than adsorbent pore diameter is independent of geometry factor of interconnecting pores, as well as surface chemical structure [66]. Herein, once the molecular size of MO is 1.2 nm and RB5 is 2.9 nm, the adsorption kinetic profiles suggest greater access to adsorption pore sites for MO dye, making the second step faster in comparison to RB5.

In order to better understand the involved adsorption mechanism for RB5 and MO dyes, the experimental data were analyzed using pseudo-first-order (PFO) and pseudo-second–order (PSO) models. The analyzed kinetic parameters are shown in Table 3.

The adjustment of the applied kinetic models was followed by the correlation coefficient (R^2^) in association to linear fit of the models. Comparing both PFO and PSO models, PSO seems to be more adequate to describe experimental results, since R^2^ values denote a good fit. Figure S3 (Supplementary Materials) shows the plots for PSO model applied for all samples. According to PSO model, the adsorption mechanism process must be directed by chemical adsorption [67]. Cao also found that azo reactive dye adsorption process, using Fe_3_O_4_/chitosan nanoparticles as adsorbent, follows a PSO kinetic model [24]. However, this model does not take into account contributions from diffusion mechanism in kinetic control. Thus, the intraparticle model was applied to provide a better understanding of the diffusion and adsorption mechanisms. Figure S4 showed the plot of $Q_{t}$ versus $t^{0.5}$ for all adsorption systems. Interestingly, it was observed that RB5 adsorption kinetics by the samples ChM GL and ECH did not exihibit a plateau, suggesting that during all contact time of adorption the process is stongly influenced by intraparticle diffusion, even at low adsorbate concentrations [4]. The linear portion of the plots should be assigned to a boundary layer sorption and the intercept value (inset in Figure S4) to a larger boundary layer effect [22]. In addition, the plots do not pass through the origin, indicating that intraparticle diffusion is not only the rate-limiting step in the adsorption process, but other kinetic processes also occurred simultaneously, contributing to the sorption mechanism [23].

#### 3.4.3. Adsorption Isotherms

**Figure 11:**
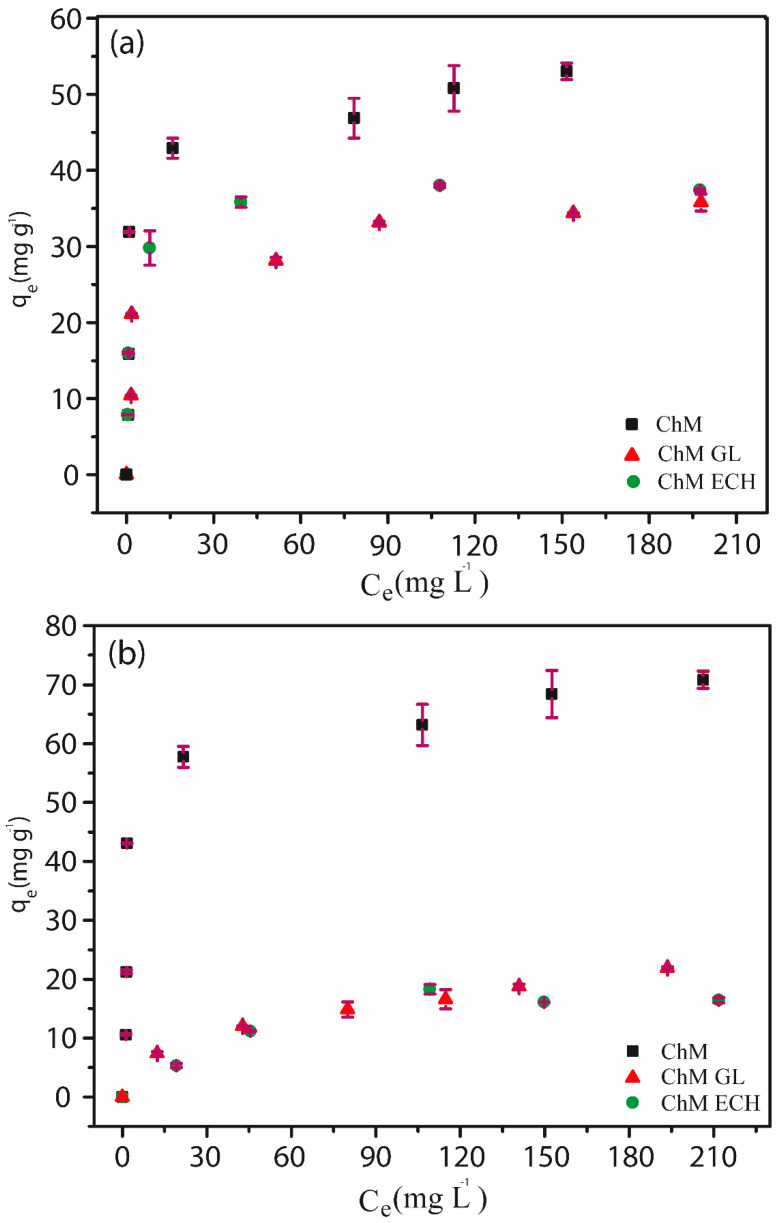
Effect of initial concentration in the adsorption capacity of (**a**) RB5 and (**b**)MO for ChM, ChM GL and ChM ECH samples.

Figure 11a,b show the influence of RB5 and MO initial concentration in the adsorption capacity of synthesized ChM NPs. For both dyes, the sample ChM did not reach an equilibrium plateau at studied concentration range. Actually, it was observed that the adsorption profile is very similar for both dyes since it showed a fast increase in the adsorption capacity by the increasing of initial concentration. This may be assigned to an increase in the adsorption sites occasioned by MNP clusters breakdown under acid medium, related to a better partial solubility of chitosan. As expected, ChM GL and ECH do not follow similar behavior, since crosslinking reactions can decrease chitosan solubility under acid medium. In addition, it was noticed that ChM sample showed a greater adsorption capacity for MO in comparison to RB5, 70.85 and 53.02 mg g^−1^, respectively, whereas adsorption capacity values found to ChM GL and ECH were 21.93 and 16.44 mg g^−1^ for MO and 35.77 and 37.39 mg g^−1^ for RB5, respectively.

Wong and coworkers [35] reported the improvement of the adsorption capacity being provided by the decrease of molecular size and the increase of dye concentration on chitosan surface, generating a greater penetration of dye molecules in the internal structure of chitosan pores. Another relevant point was investigated by Zhou and coworkers [68], which evaluated the adsorption capacity regarding dye molecular size and different amounts of sulfonic groups. They observed that the dye with highest number of sulfonic groups was mostly adsorbed on the adsorbent surface, suggesting an adsorption mechanism controlled by electrostatic interaction.

Thus, according to the literature for ChM sample, our results suggest that the molecular size seems to be a dominant effect, since a lower adsorption capacity was observed for RB5, even with three more sulfonic groups than MO dye. Interestingly, for modified samples, N_2_ adsorption isotherms showed a higher number of pores with size around <3 nm, which may provide a better access to RB5 molecules in their internal pores in comparison to ChM NPs. Therefore, for both dyes in ChM GL and ECH samples, this increase in pore size together with similar adsorption capacity suggests a strong influence of the molecular size effect in the adsorption process. However, it is important to notice that electrostatic interactions must govern dyes adsorption mechanisms, promoting a smaller adsorption capacity for modified samples. In general, surface-modified chitosan-based materials decrease their adsorption capacity due to the decreasing of accessibility to internal sites or blockade of adsorption sites [69]. The maximum adsorption capacity of chitosan-based adsorbents for RB5 and MO under similar experimental conditions is shown in Table S1. The synthesized nanocomposites in this work do not have a high adsorption capacity in comparison to other materials. However, this profile can be due to small percentage of chitosan present in the nanocomposite, as already discussed by TGA analyses.

The correlation of isotherms data to each theoretical or empirical equation is essential for a practical and operational planning of the adsorption system. Several mathematical models were used to describe different adsorption processes and the establishment of most appropriate correlation for equilibrium curves must be done. Herein, in this work, mathematical models such as Langmuir, Freundlich, Redlich–Peterson and Temkin were applied to better discuss obtained adsorption results. Table 4 summarizes all parameters obtained from the fit applied to unmodified and modified samples for both RB5 and MO dyes. Moreover, Figures S5 and S6 (Supplementary Materials) show all fits applied to experimental data.

In general, all applied models showed a value of Adj. R^2^ lower than 0.99, except for the sample ChM GL for MO adsorption. ChM GL obtained a satisfactory Adj. R^2^ when adjusted to the Freundlich model, suggesting that the adsorption mechanism of MO should be through multilayers. However, once ChM GL was applied as adsorbent of RB5, the Temkin model better described the adsorption mechanism. For ChM ECH, according to all results observed in Table 4, the Langmuir model greatest described the MO adsorption process, whereas for RB5 is the Redlich–Peterson model. In addition, β values suggest that this process is approaching to Langmuir model at low concentration.

Interestingly, for ChM, both MO and RB5 adsorption process obtained low values of Adj. R^2^—and comparing applied models—Langmuir greatest described the adsorption processes. Kyzas [70] investigated the adsorption of dyes on chitosan surface and observed that, at low concentrations of adsorbate, the adsorption process occurred by stronger electrostatic interaction and the equilibrium condition is quickly reached. Herein, the fast adsorption may be due to low dye concentration used in the assays, suggesting a monolayer formation, which is in accordance with adjustments of Langmuir model. It is also important to notice that, for both ChM GL and ECH samples, different isotherms were needed to evaluate the adsorption process, whereas ChM was just applied to the Langmuir model for both dyes. This should be assigned to the chemical modification of chitosan molecules occasioned by crosslinking reaction, which also modifies pore volume and surface area of the samples.

**Figure 12:**
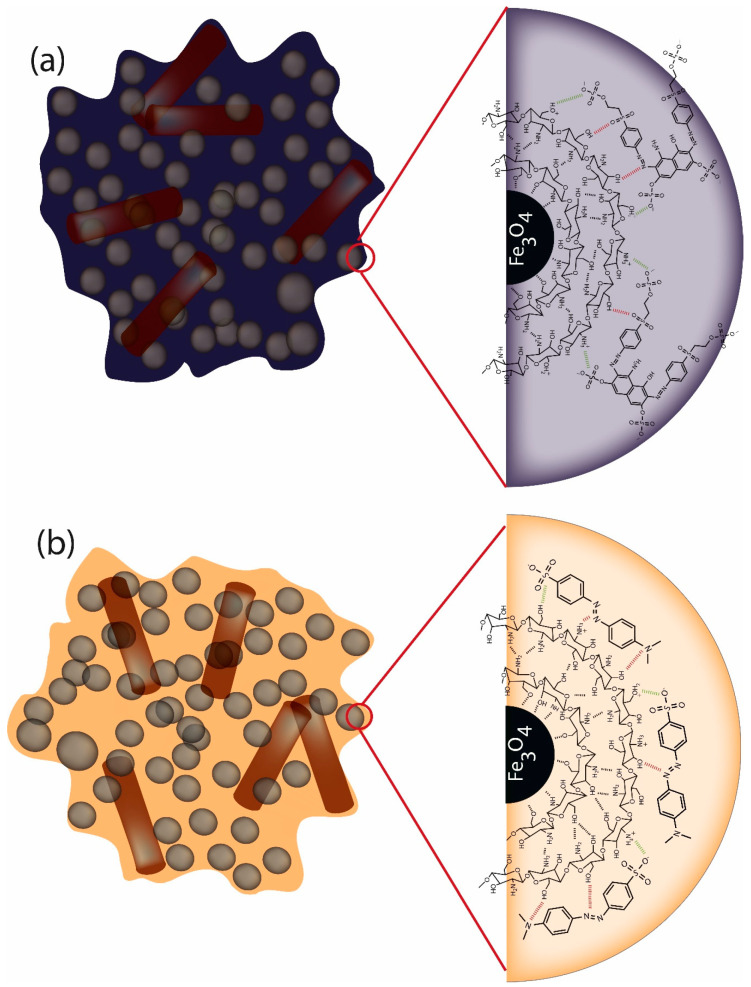
Schematic illustration of adsorption possibilities for ChM sample: (**a**) RB5 and (**b**) MO.

Therefore, based on overall results obtained in the adsorption process for both RB5 and MO dyes, both electrostatic and hydrogen-bonding interactions can be considered. Herein, in Figure 12 is shown a proposal of the interaction possibilities between dye molecules and ChM sample. For this proposal, we assumed the pH 4 for adsorption systems, which is lower than the pKa of chitosan (6.5). Indeed, ChM nanocomposites should have a surface rich in protonated amino and hydroxyl groups. In addition, since RB5 and MO have sulfonic groups, under pH 4 a strong ionic interaction must be observed due to chitosan on MNPs surface. Thus, considering that RB5 has low penetration in chitosan network, Figure 12 suggests that the adsorption process occurred by a dye monolayer formation on ChM surface. In addition, for this sample, the Langmuir model was the one that better describe the isotherms results.

## 4. Conclusions

In summary, magnetic chitosan nanocomposite was successfully synthesized through a well-established ultrafast strategy under US irradiation. All modified and unmodified ChM nanocomposites presented good crystallinity, high M_s_ ranging from 44 to 57 emu·g^−1^ and high surface area. In addition, the adopted methodology provided MNPs with nanorod and spherical type-morphologies. Regarding adsorption assays, the chosen anionic RB5 and MO dyes were analyzed at different experimental conditions on nanocomposites surface. As expected, the adsorption capacity of both dyes was significantly affected by pH medium, suggesting that electrostatic interactions had great influence on adsorption mechanisms. Indeed, kinetic study showed a relevant dependence between molecular size of adsorbates and the adsorption process. For instance, adsorption capacity values for unmodified ChM sample were approximately 50 and 70 mg·g^−1^ for RB5 and MO, respectively, i.e., the adsorption process was mainly driven through dye molecular size itself. Considering equilibrium conditions, ChM ECH sample reached the equilibrium faster in comparison to ChM and ChM GL. Herein, according to isotherm models, different profiles of adsorption mechanisms were found. Nevertheless, all synthesized nanocomposites seem to be influenced by electrostatic interactions. In this sense, amino-based superparamagnetic materials may present a susceptibility to natural organic matter (NOM), mainly humic acid that could compete by the adsorption sites, decreasing the efficiency of the adsorbent in the removal of azo dyes. However, since textile effluents may show low levels of NOM concentration, these ChM nanocomposites show great potential to be applied as adsorption system in textile wastewater.

## Figures and Tables

**Table 1:** Crystallographic data obtained from Rietveld refinement.

Samples	R_wp_ (%)	χ^2^	a, b and c (Å)	D (nm)
Fe_3_O_4_	11.73	1.04	8.3625	11.60 ± 0.11
ChM	14.75	0.94	8.3655	13.33 ± 0.47
ChM GL	12.39	1.14	8.3533	11.92 ± 0.16
ChM ECH	11.80	1.02	8.3548	11.74 ± 1.73

**Table 2:** Assignment of spectral peaks (Fe, C, O and N) based on binding energies and atomic fraction.

Element	Binding Energy (eV)	Atomic Fraction (%)	Assignments
C 1s	284.9	34.36	C–C
	286.5		C–OH; C–NH_2_
	288.2		O–C–O
N 1s	399.6	3.88	NH_2_
	400.6		NH_2_---O
	402.0		NH_2_---Fe
O 1s	529.8	51.7	Fe–O
	530.9		–OH
	533.0		–OH---Fe
Fe 2p_3/2_	710.1	10.6	Fe^2+^ Oct
	711.3		Fe^3+^ Thd
	713.3		Fe^3+^ Oct
Fe 2p_1/2_	723.4		Fe^2+^ Oct
	724.4		Fe^3+^ Oct
	727.7		Fe^3+^ Thd

**Table 3:** Kinetic parameters for RB5 and MO dyes adsorptions.

Reactive Black 5									
Model	Pseudo-first-order			Pseudo-second–order			Intraparticle diffusion		
	R^2^	q_e_ (mg g^−1^)	k_1_ (min^−1^)	R^2^	q_e_ (mg g^−1^)	k_2_ (g mg^−1^ min^−1^)	K_p_ (g mg^−1^ t^−0.5^)	C	R^2^
ChM	0.9770	33.420	0.14675	0.9998	33.200	0.01180	7.7541	1.4071	0.9682
ChM GL	0.9714	23.816	0.4779	0.9954	24.485	0.00270	1.7490	4.759	0.9976
ChM ECH	0.9532	12.062	0.00673	0.9870	35.211	0.00206	0.6260	19.949	0.9856
**Methyl orange**									
ChM	0.9828	8.682	0.1045	0.9999	27.762	0.03858	3.2495	15.2461	0.9890
ChM GL	–	–	–	0.9994	18.175	−0.05491	2.0206	13.5764	0.9786
ChM ECH	0.9798	15.807	0.8833	0.9990	26.055	0.02207	4.2461	8.5636	0.8749

**Table 4:** Parameters obtained from the fits of Langmuir, Freundlich, Redlich–Peterson and Temkin isotherms for both RB5 and MO dyes.

Dye	Reactive Black 5				Methyl Orange			
Langmuir	Adj. R^2^	q_m_ (mg g^−1^)	K_a_ (L mg^−1^)		Adj. R^2^	q_m_ (mg g^−1^)	K_a_ (L mg^−1^)	
ChM	0.913	50.004	0.820		0.903	68.004	0.402	
ChM ECH	0.978	36.944	0.932		0.989	20.386	0.0229	
ChM GL	0.923	33.630	0.523		0.964	24.647	0.0231	
Freundlich	Adj. R^2^	K_f_ (L g^−1^)	n		Adj. R^2^	K_f_ (L g^−1^)	n	
ChM	0.873	21.438	5.390		0.861	25.652	5.071	
ChM ECH	0.907	17.346	6.053		0.957	2.248	2.617	
ChM GL	0.938	14.531	5.774		0.996	2.633	2.515	
Redlich–Peterson	Adj. R^2^	K_rp_ (L mg^−1^)	A_rp_ (L mg^−1^)^β^	β	Adj. R^2^	K_rp_ (L mg^−1^)	A_rp_ (L mg^−1^)^β^	β
ChM	0.900	47.918	1.167	0.955	0.886	30.964	0.545	0.962
ChM ECH	0.984	40.674	1.302	0.960	0.973	0.876	0.154	0.776
ChM GL	0.926	48.866	2.699	0.871	0.986	1.053	0.154	0.768
Temkin	Adj. R^2^	K_T_ (L mg^−1^)	B (J mol^−1^)		Adj. R^2^	K_T_ (L mg^−1^)	B (J mol^−1^)	
ChM	0.899	25.003	6.4895		0.887	10.636	9.3778	
ChM ECH	0.953	35.964	4.6139		0.983	0.194	4.6397	
ChM GL	0.950	25.201	4.1678		0.978	0.299	4.9853	

## Supplementary Materials

The following are available online at https://www.mdpi.com/2079-4991/10/6/1194/s1, Figure S1: R_L_ value for (**a**) RB5 and (**b**) MO adsorption from the Langmuir isotherm; Figure S2: Zeta potential of ChM nanocomposite at different pH levels; Figure S3: Pseudo-second order plots for (**a**) reactive black 5 and (**b**) methyl orange adsorption onto nanocomposites; Figure S4: (**a**), (**b**) and (**c**) show the intraparticle diffusion plots for sorption of MO; (**d**), (**e**) and (**f**) show the intraparticle diffusion plots for sorption of RB5; Figure S5: Fits of applied isotherms models to the experimental data for adsorption of reactive black 5 onto (**a**) ChM, (**b**) ChM ECH and (**c**) ChM GL; Figure S6: Fits of applied isotherms models to the experimental data for adsorption of methyl orange onto (**a**) ChM, (**b**) ChM GL and (**c**) ChM ECH. Table S1: Comparison of the maximum adsorption capacity of ChM, ChM GL and ChM ECH to different modified chitosan adsorbents in the literature.
