# Supplementary material for: Magnetic Porous Controlled Fe3O4–Chitosan Nanostructure: An Ecofriendly Adsorbent for Efficient Removal of Azo Dyes
Source: Nanomaterials (Basel). 2020 Jun 19;10(6):1194. doi: 10.3390/nano10061194 (PMC7353100; doi:10.3390/nano10061194)
Supplement: Supplementary file 1 [file nanomaterials-10-01194-s001.pdf]

Supporting Information for

Magnetic Porous Controlled Fe_3O_4–Chitosan Nanostructure: An Ecofriendly Adsorbent for Efficient Removal of Azo Dyes

Tiago M. Freire [1], Lillian M. U. D. Fechine [1], Danilo C. Queiroz [2], Rafael M. Freire [3], Juliano C. Denardin [4], Nágila M. P. S. Ricardo [2], Thaina N. B. Rodrigues [5], Diego R. Gondim [5], Ivanildo J. S. Junior [5] and Pierre B. A. Fechine [1,*]

[1] Group of Chemistry of Advanced Materials (GQMat)—Department of Analytical Chemistry and Physical-Chemistry, Federal University of Ceará—UFC, Campus do Pici, CP 12100, CEP 60451-970 Fortaleza, Brazil; tiagomf@ufc.br (T.M.F.); lmudutra@hotmail.com (L.M.U.D.F.)

[2] Department of Organic and Inorganic Chemistry, Federal University of Ceará—UFC, Campus do Pici, CP 12100, CEP 60451-970 Fortaleza, Brazil; daniloqueiroz46@gmail.com (D.C.Q.); naricard@ufc.br (N.M.P.S.R.)

[3] Institute of Applied Chemical Sciences, Universidad Autónoma de Chile, 8910060 Santiago, Chile; rafael.m.freire@gmail.com

[4] Department of Physical/CEDENNA, University of Santiago de Chile, USACH, Av. Ecuador 3493, 9170020 Santiago, Chile; juliano.denardin@usach.cl

[5] Department of Chemical Engineering, Federal University of Ceará—UFC, Campus do Pici, CP 12100, CEP 60451-970 Fortaleza, Brazil; thaina.nobre@hotmail.com (T.N.B.R.); diegoromao19@yahoo.com.br (D.R.G.); ivanildo@gpsa.ufc.br (I.J.S.J.)

* Correspondence: fechine@ufc.br; Tel.: +55-(85)33669047

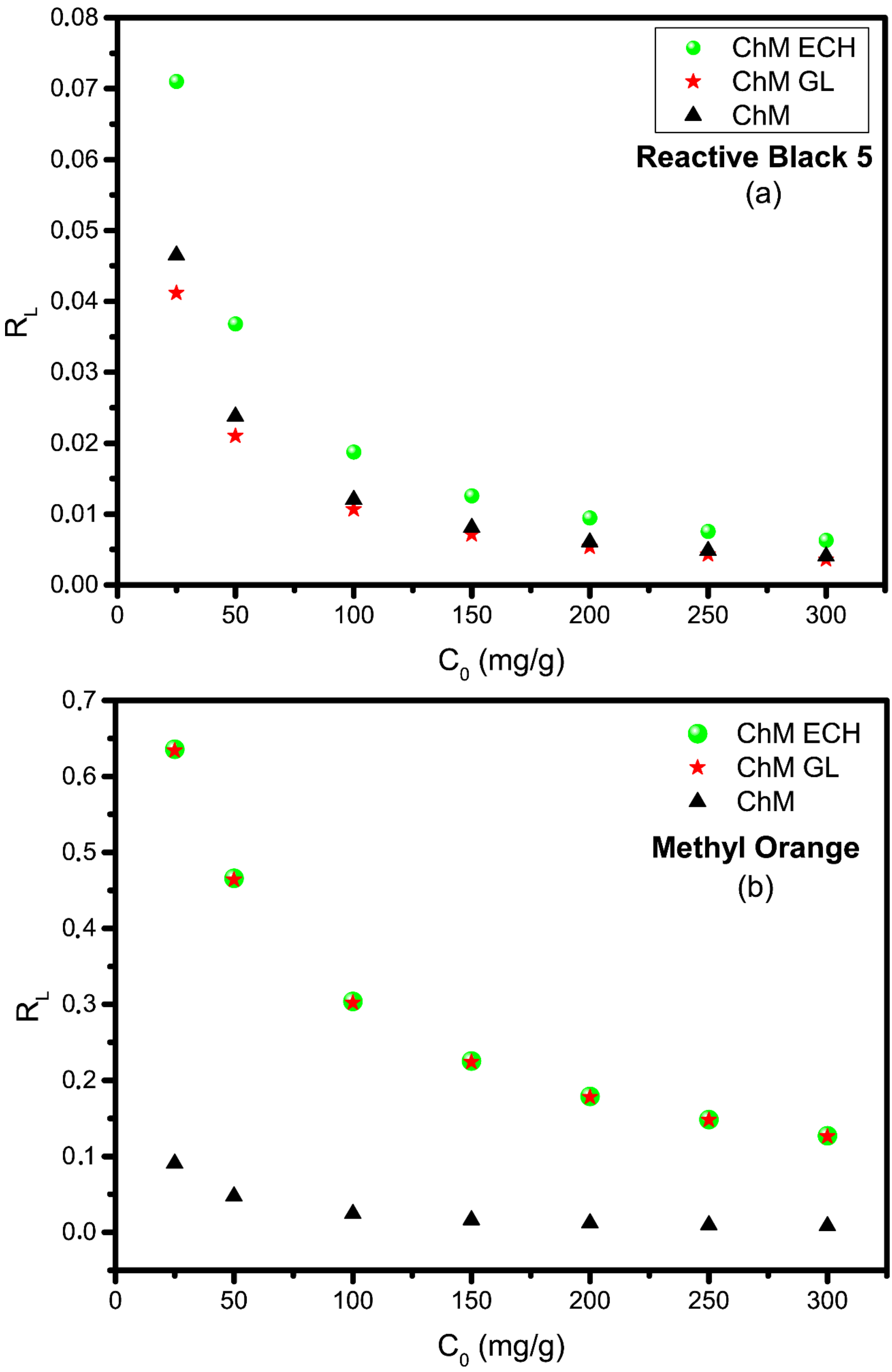

Figure S1: R_L value for (a) RB5 and (b) MO adsorption from the Langmuir isotherm.

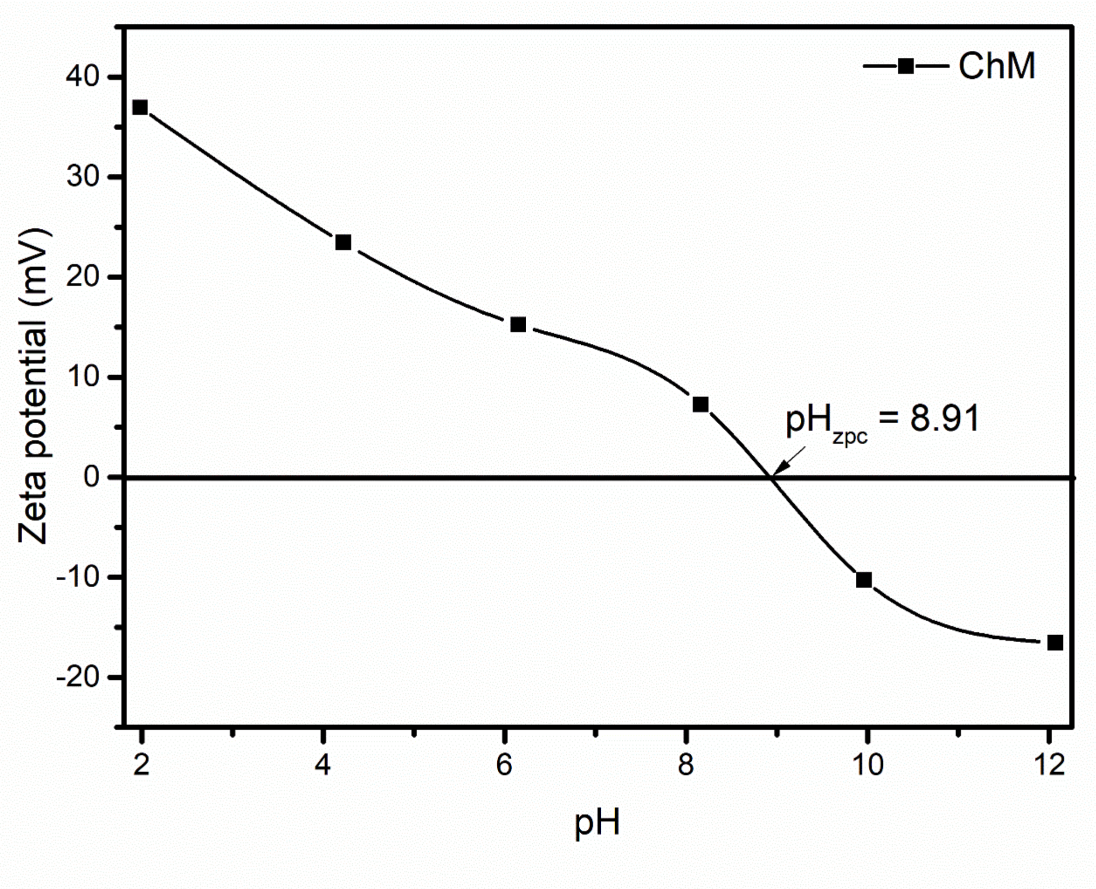

Figure S2: Zeta potential of ChM nanocomposite at different pH levels.

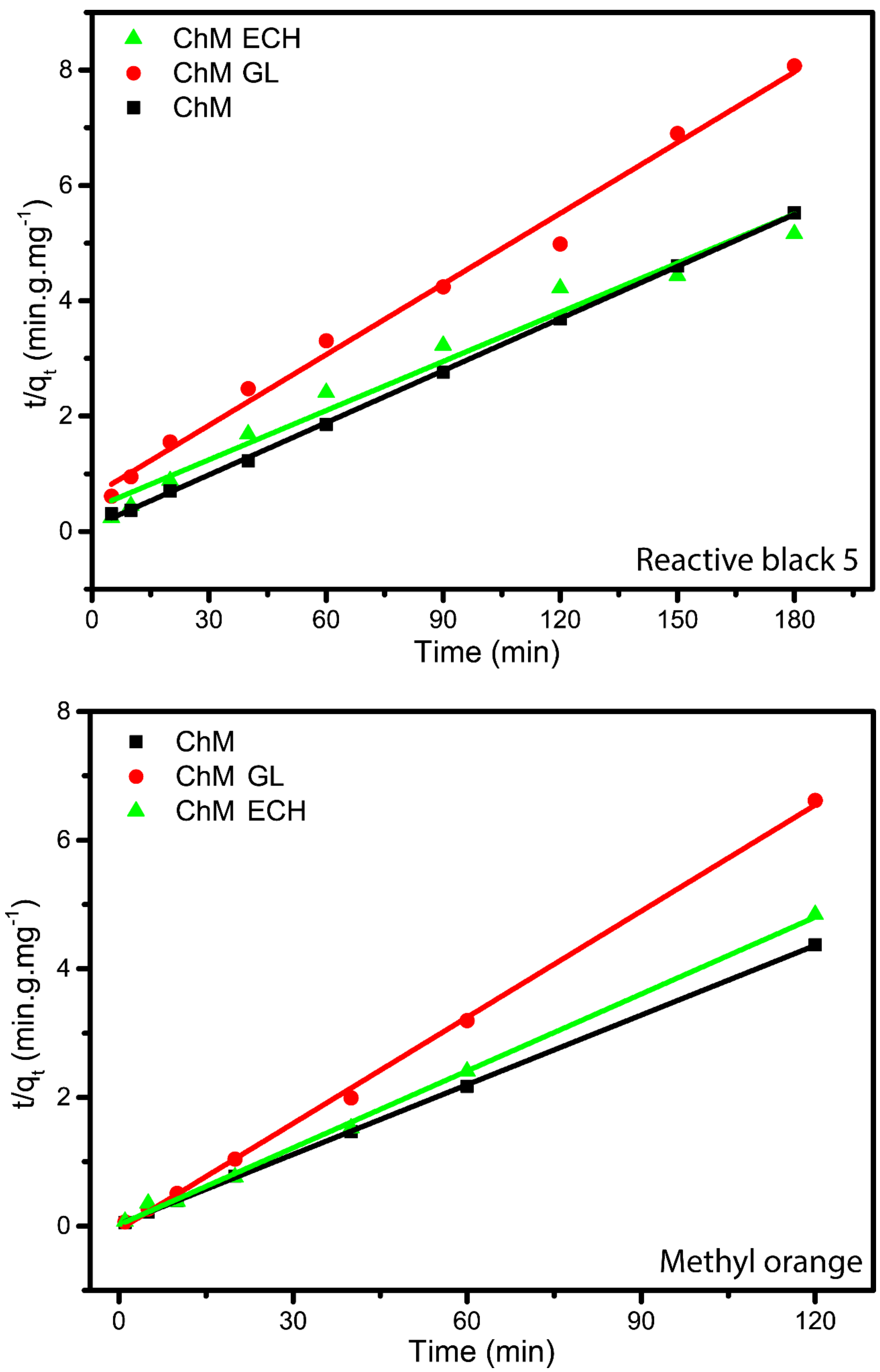

Figure S3: Pseudo-second order plots for (a) reactive black 5 and (b) methyl orange adsorption onto nanocomposites.

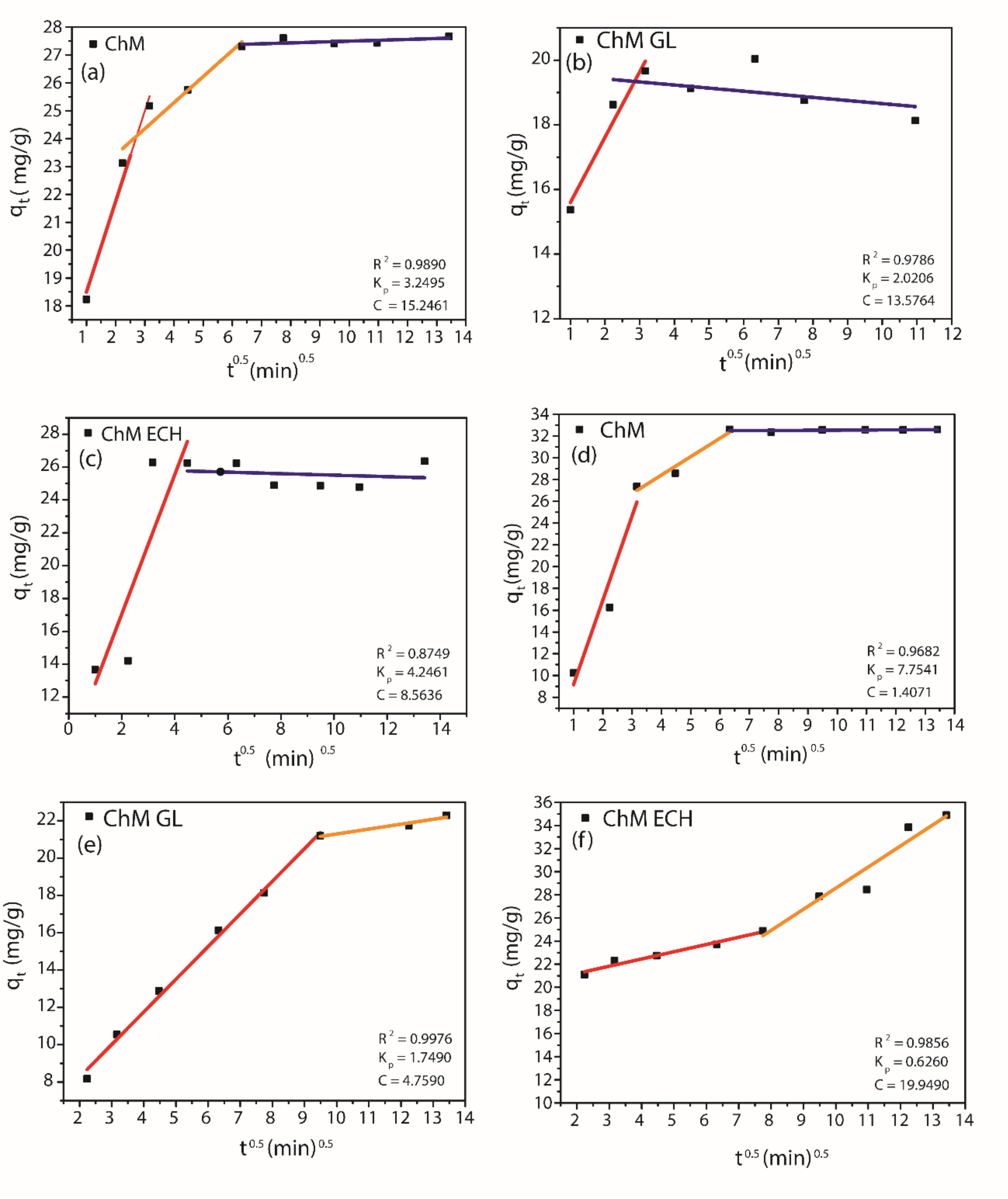

Figure S4: (a), (b), and (c) show the intraparticle diffusion plots for sorption of MO; (d), (e), and (f) show the intraparticle diffusion plots for sorption of RB5.

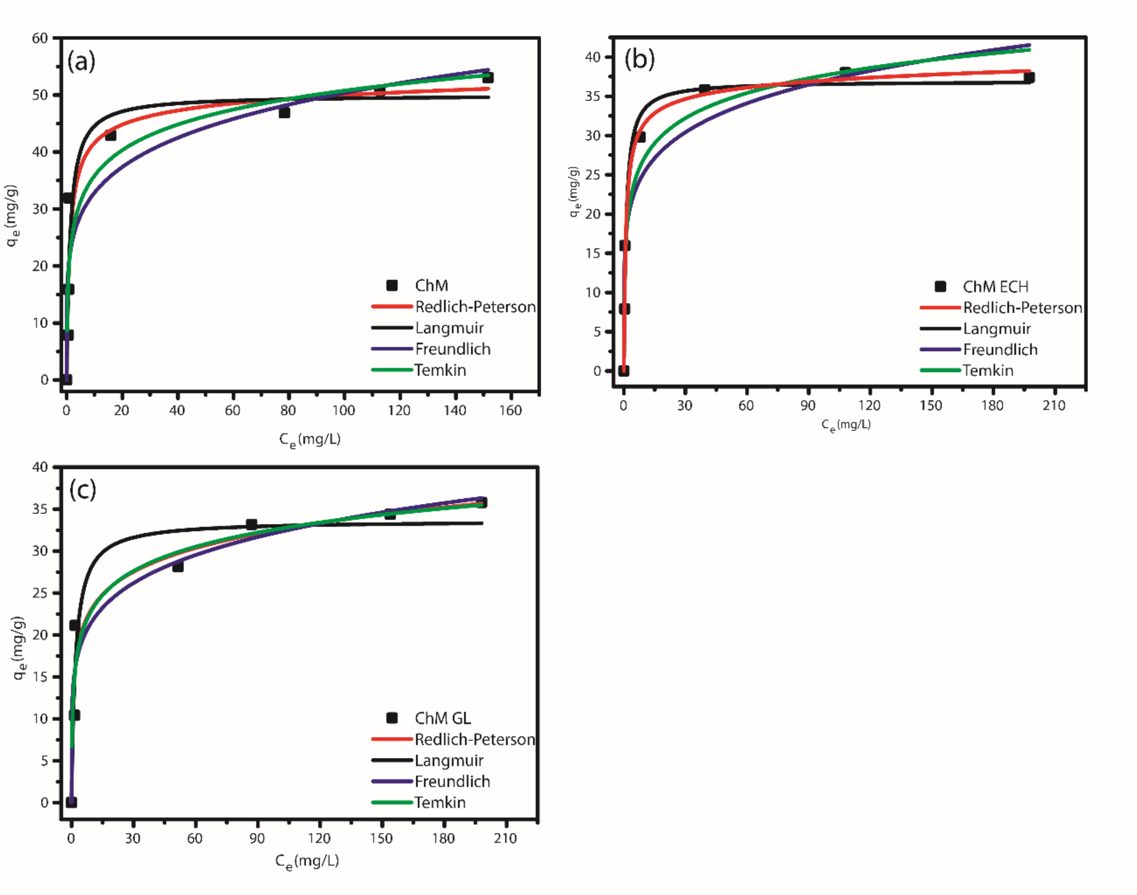

Figure S5: Fits of applied isotherms models to the experimental data for adsorption of reactive black 5 onto (a) ChM, (b) ChM ECH and (c) ChM GL.

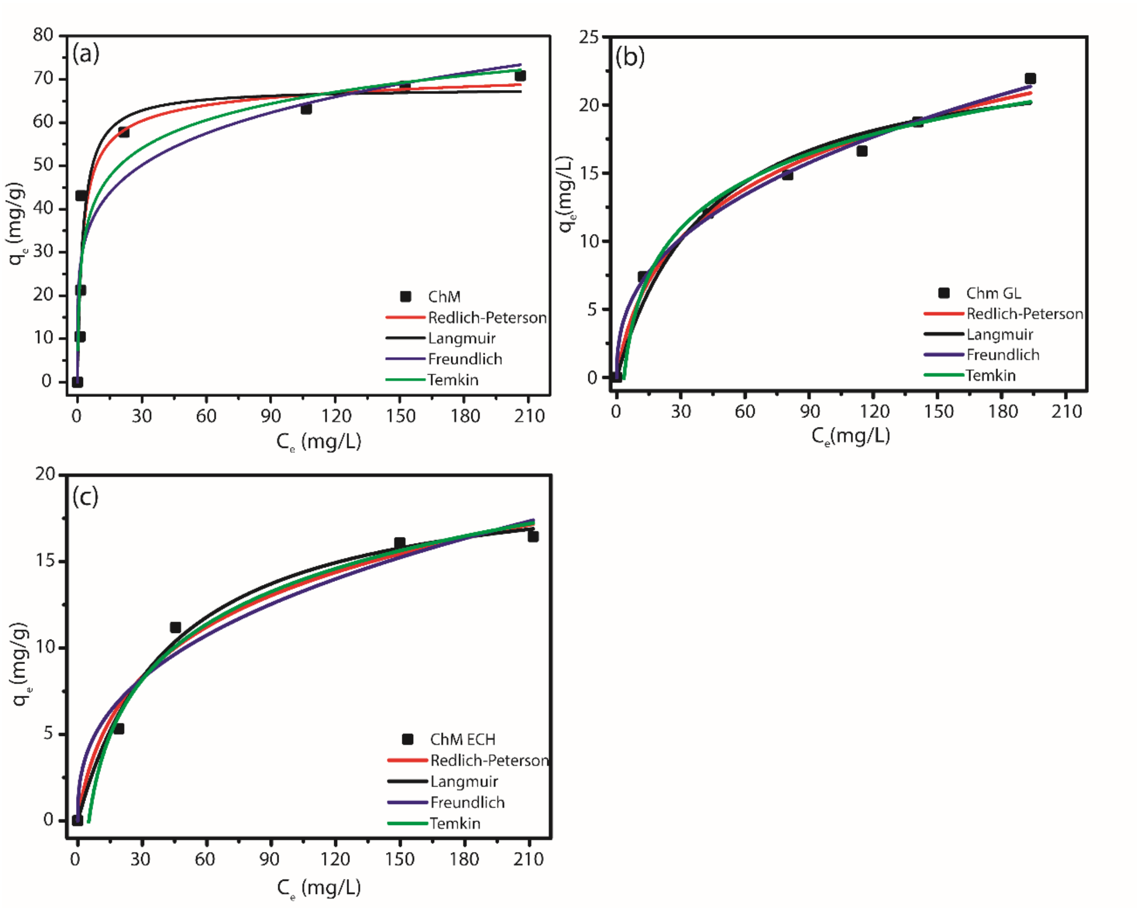

Figure S6: Fits of applied isotherms models to the experimental data for adsorption of methyl orange onto (a) ChM, (b) ChM GL and (c) ChM ECH.

Table S1. Comparison of the maximum adsorption capacity of ChM, ChM GL and ChM ECH to different modified chitosan adsorbents in the literature.

**Adsorbent**	**Adsorption Capacity (mg g $^{-1}$)**		**Reference**
	RB5	MO	
γ-Fe_2O_3/chitosan	-	29.41	[1]
Fe3O4-chitosan- L-arginine	-	338.98	[2]
m-CS/Fe3O4/MIL-101	-	117	[3]
chitosan/Al2O3/magnetite	-	417	
Graphene Oxide/Fe_3O_4	391	-	[4]
Eichhornia crassipes/chitosan	0.606	-	[5]
Magnetic chitosan - glutaraldehyde	357.10	-	[6]
Modified chitosan-pandan	169.49	-	[7]
ChM	53.02	70.85	
ChM GL	35.77	21.93	This study
ChM ECH	37.39	16.44	

Reference

[1] R. Jiang, Y.-Q. Fu, H.-Y. Zhu, J. Yao, L. Xiao, Removal of methyl orange from aqueous solutions by magnetic maghemite/chitosan nanocomposite films: Adsorption kinetics and equilibrium, Journal of Applied Polymer Science 125(S2) (2012) E540-E549.

[2] S. Guo, J. Zhang, X. Li, F. Zhang, X. Zhu, Fe3O4-CS-L: a magnetic core-shell nano adsorbent for highly efficient methyl orange adsorption, Water Science and Technology 77(3) (2017) 628-637.

[3] L. Liu, J. Ge, L.-T. Yang, X. Jiang, L.-G. Qiu, Facile preparation of chitosan enwrapping Fe3O4 nanoparticles and MIL-101(Cr) magnetic composites for enhanced methyl orange adsorption, Journal of Porous Materials 23(5) (2016) 1363-1372.

[4] N.A. Travlou, G.Z. Kyzas, N.K. Lazaridis, E.A. Deliyanni, Functionalization of Graphite Oxide with Magnetic Chitosan for the Preparation of a Nanocomposite Dye Adsorbent, Langmuir 29(5) (2013) 1657-1668.

[5] M.M. El-Zawahry, F. Abdelghaffar, R.A. Abdelghaffar, A.G. Hassabo, Equilibrium and kinetic models on the adsorption of Reactive Black 5 from aqueous solution using Eichhornia crassipes/chitosan composite, Carbohydrate Polymers 136 (2016) 507-515.

[6] B. Tural, T. Tarhan, S. Tural, removal of reactive black 5 (rb5) from aqueous solutionby cross-linked magnetic biosorbent, (2017).

[7] F.A. Razmi, N. Ngadi, S. Wong, I.M. Inuwa, L.A. Opotu, Kinetics, thermodynamics, isotherm and regeneration analysis of chitosan modified pandan adsorbent, Journal of Cleaner Production 231 (2019) 98-109.
